# Microfluidic chip as a promising evaluation method in assisted reproduction: A systematic review

**DOI:** 10.1002/btm2.10625

**Published:** 2023-11-24

**Authors:** Tong Wu, Yangyang Wu, Jinfeng Yan, Jinjin Zhang, Shixuan Wang

**Affiliations:** ^1^ National Clinical Research Center for Obstetrical and Gynecological Diseases Tongji Hospital, Tongji Medical College, Huazhong University of Science and Technology Wuhan China; ^2^ Key Laboratory of Cancer Invasion and Metastasis, Ministry of Education Tongji Hospital, Tongji Medical College, Huazhong University of Science and Technology Wuhan China; ^3^ Department of Obstetrics and Gynecology Tongji Hospital, Tongji Medical College, Huazhong University of Science and Technology Wuhan China; ^4^ College of Animal Science and Technology Sichuan Agricultural University Ya'an Sichuan China; ^5^ School of Materials Science and Engineering Huazhong University of Science and Technology Wuhan China

**Keywords:** assisted reproductive technology, evaluation, female reproduction, microfluidic chip, microfluidics

## Abstract

The aim of assisted reproductive technology (ART) is to select the high‐quality sperm, oocytes, and embryos, and finally achieve a successful pregnancy. However, functional evaluation is hindered by intra‐ and inter‐operator variability. Microfluidic chips emerge as the one of the most powerful tools to analyze biological samples for reduced size, precise control, and flexible extension. Herein, a systematic search was conducted in PubMed, Scopus, Web of Science, ScienceDirect, and IEEE Xplore databases until March 2023. We displayed and prospected all detection strategies based on microfluidics in the ART field. After full‐text screening, 71 studies were identified as eligible for inclusion. The percentages of human and mouse studies equaled with 31.5%. The prominent country in terms of publication number was the USA (*n* = 13). Polydimethylsiloxane (*n* = 49) and soft lithography (*n* = 28) were the most commonly used material and fabrication method, respectively. All articles were classified into three types: sperm (*n* = 38), oocytes (*n* = 20), and embryos (*n* = 13). The assessment contents included motility, counting, mechanics, permeability, impedance, secretion, oxygen consumption, and metabolism. Collectively, the microfluidic chip technology facilitates more efficient, accurate, and objective evaluation in ART. It can even be combined with artificial intelligence to assist the daily activities of embryologists. More well‐designed clinical studies and affordable integrated microfluidic chips are needed to validate the safety, efficacy, and reproducibility.

Trial registration: The protocol was registered in the Open Science Frame REGISTRIES (identification: osf.io/6rv4a).

**Translational Impact Statement**

Microfluidic chips enable a more comprehensive, precise, and point‐of‐care pipeline to analyze reproductive samples and promote assisted reproductive technologies. They provide novel cues on cell motility, morphological alteration, cell mechanics, impedance, secretion, oxygen consumption, and metabolism at single cell level for sperm, oocytes, and embryos. Moreover, artificial intelligence can be integrated into the microfluidic system to assist embryologists in their daily activities and help patients achieve their goal of having a healthy baby.

## 1 INTRODUCTION

**FIGURE 1:**
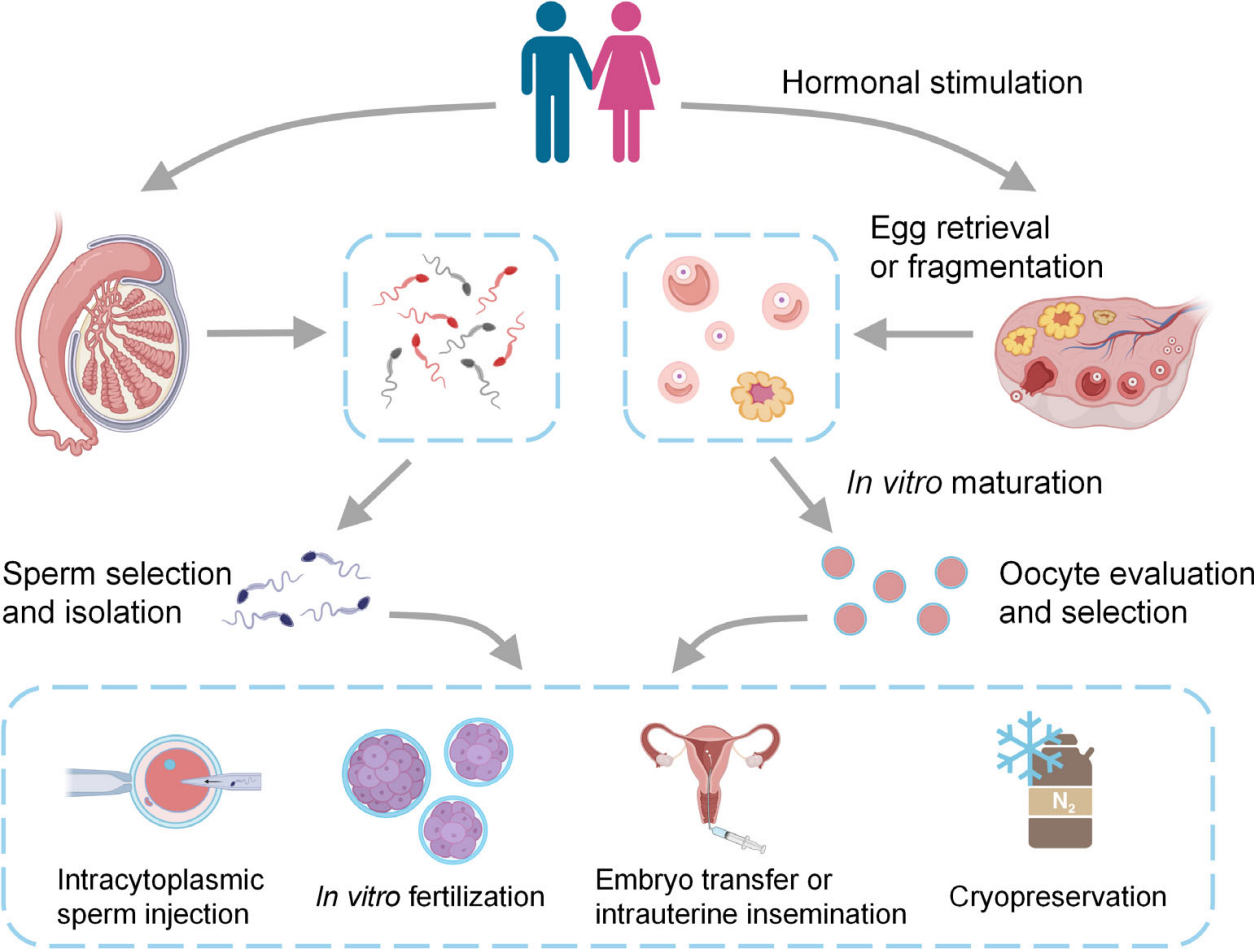
Overview of conventional assisted reproductive technology procedures. Gametes (sperm/oocytes) are directly obtained from males and females or extracted through in vitro manipulations. Matured oocytes are incubated with competent sperm to produce embryos in a process called in vitro fertilization. Fertilization and ongoing pregnancies can also be achieved for sperm that might not be competent using intracytoplasmic sperm injection. In vitro‐produced embryos are selected and transferred to the uterus of recipients. Alternatively, gametes, embryos, ovarian and testicular tissues can be cryopreserved and stored in biobanks.

Infertility is a highly prevalent global disorder affecting 8%–15% of reproductive‐age couples worldwide and is getting worse due to environmental exposure, stress, irregular lifestyle, and delayed childbearing.^1^, ^2^, ^3^ Consequently, the mental stress and financial strain attributed to infertility have become non‐neglected social issues in most countries.^4^ Addressing fertility problems for couples seeking a pregnancy could significantly alleviate population collapse and social burden.^5^, ^6^ Assisted reproductive technologies (ARTs) have come into existence for over 50 years and account for 2% of all births nowadays.^3^ ARTs encompass various medical treatments and procedures related to gamete and embryo handling, including hormonal stimulation of the ovaries, egg retrieval, in vitro maturation, in vitro fertilization (IVF), intracytoplasmic sperm injection (ICSI), in vitro embryo culture, embryo transfer, gamete and embryo cryopreservation, etc. The samples must be carefully evaluated and selected in each step to increase the success rate (Figure 1).^7^ That is to say, the quality of sperm, oocytes, and embryos determines the success of a pregnancy. Moreover, embryo quality requirements were raised since the elective single‐embryo transfer has been widely adopted to reduce the risk of multifetal gestation.^8^ Given these circumstances, there is a compelling motivation to discern competent gametes and embryos to foster ART development.^9^

Current assessment criteria for gametes and embryos rely mainly on morphological observations. A plethora of characteristics have been included to rank or score human samples.^10^, ^11^ For instance, healthy oocytes should exhibit an intact plasma membrane and arranged chromosomes and spindles, while aged oocytes display chromosomal anomalies, partial exocytosis, and zona pellucida (ZP) alterations.^12^ In the case of sperm, subtle nucleus malformations in shape, size, and vacuoles might not be noticed but still decrease the quality of the paternal contributions to embryos.^13^ Variations in the acrosome, neck, and tail of spermatozoa have similar effects.^14^ Indicators for embryo viability include a morphological scoring system, cleavage rate, nutrient uptake capacity, and so on.^15^ However, despite all these detailed and standard features, the subjective judgment of embryologists varies even within the same clinic.^16^, ^17^ Additionally, manual operations require highly skilled and experienced assessors and tedious, time‐consuming work. The development of biomedical and engineering technologies could enable a more comprehensive, precise, and point‐of‐care pipeline to analyze reproductive samples and advance ARTs.

Microfluidics is one of the most powerful and attractive methods to analyze biological samples as it delivers the following benefits: (i) reducing the number of reagents, solvents, and samples; (ii) lowering the risk of contamination from the external environment; (iii) allowing long‐term culture, automatic delivery, and high throughput application.^18^ Traditional assessment procedures concerning oocytes and embryos involve transferring samples between dishes at specified times and positions, resulting in an altered culture environment (pH, CO_2_ concentration, and temperature) and causing metabolic stress.^19^ Microfluidic devices coupled with external equipment can maintain cells under constant culture conditions, minimize the handling and observation time, and retain germplasm viability. Microfluidics has been applied to assess sperm motility for over two decades, gradually occupying an important position in the field.^20^ More advanced microfluidic devices have been fabricated to simultaneously detect four hormones (beta‐human chorionic gonadotropin, follicle‐stimulating hormone, luteinizing hormone, and prolactin) with high sensitivity.^21^, ^22^ Several reviews have discussed the use of microfluidics for sperm sorting,^23^, ^24^ biomechanical analysis of oocyte and embryo,^25^ and drug screening,^26^ mainly focusing on cell manipulation and drug discovery. However, studies conveying comprehensive information about the assessment methods in ARTs are lacking. Nowadays, the microfluidics have been applied in detecting the motility, mechanics, structure, and oxygen consumption of gametes or embryos. A well‐conducted systematic review of the frontiers of microfluidics could summarize the current status in ART, improve its reliability, and inspire novel perspectives for future development.

We systematically searched five databases based on the Preferred Reporting Items for Systematic Reviews and Meta‐Analyses (PRISMA) guidelines using a set of selected keywords to determine the use of microfluidics assessment methods in ART. Microfluidics could contribute to a more accurate evaluation of gametes and embryos than the traditional methods and hold a wide future potential.

## 2 METHODS

### 2.1 Information sources

This systematic review was performed according to PRISMA protocol.^27^ The protocol was registered in the Open Science Frame REGISTRIES (identification: osf.io/6rv4a). A systematic search was conducted in five electronic medical databases: PubMed, Scopus, Web of Science, ScienceDirect and IEEE Xplore. The search was not limited by language. The date is limited to March 2023.

### 2.2 Search strategy

Search terms were based on a PICO (population, intervention, comparison, and outcome) framework: (P) All models that relevant with ART. (I) Microfluidics. (O) Evaluation outcome. To maximally cover the relevant literature, we used the following search keywords: (assisted reproductive technology OR in vitro fertilization OR clomiphene citrate OR superovulation OR gamete intrafallopian transfer OR in vivo fertilization OR zygote intrafallopian transfer OR artificial insemination OR cryopreservation OR intracytoplasmic sperm donation OR embryo OR oocyte OR sperm OR infertility therapy OR assisted reproduction) AND (microfluidic OR chip) AND (monitor OR assay OR sensor OR analyze OR biochemical OR screen OR biomarker) (Table S1). Additional studies were identified by manually searching the reference lists of the selected articles and complementary reviews.

### 2.3 Eligibility criteria

Articles were selected for inclusion in the systematic review if they fulfilled the following criteria: (1) original, rigorous, and accessible peer‐reviewed work until March 2023; (2) use of microfluidic platforms in ART studies; (3) use of cell or tissues from mammals.

Studies were excluded for the following reasons: (1) review articles, conference/meeting papers, commentaries, communications, perspectives, and editorials; (2) computational simulations without cells or germ cells; (3) observations not intended for ART; (4) Ex vivo models that only focus on sorting, culture, manipulation, and cryopreservation; (5) non‐mammalians. Different original articles using chips of the same type by the same group were defined as repeated records and the latest article was retained.

### 2.4 Study selection

Two authors (T.W. and Y.Y.W.) independently searched the electronic medical databases and selected studies based on eligibility criteria. The titles and abstracts were manually examined. The reasons for exclusion and inclusion for all articles were recorded as well. Discrepancies between selected studies from both authors were discussed in a consensus meeting with the senior author (S.X.W.) giving a binding verdict.

### 2.5 Data extraction

Data extraction included the titles, authors, year of publication, and keywords. Outcomes of interest included the animal species, fabrication methods, materials, cellular types, groups, and main findings. The above items were carefully revised by J.J.Z. and S.X.W.

### 2.6 Visualization and bibliometric analysis

All keywords (titles, abstracts, and authors' keywords) of the eligible studies were used to analyze the occurrence frequency by VOSviewer (Version 1.6.18). Keywords with the same meaning were integrated into a single keyword. For instance, “chip,” “microfluidic chip,” and “microfluidics” were combined into “microfluidic chip.” The word cloud was visualized by the OmicShare online tool according the frequency of each keyword (https://www.omicshare.com/toolsomicsha).

## 3 RESULTS

### 3.1 Study selection

**FIGURE 2:**
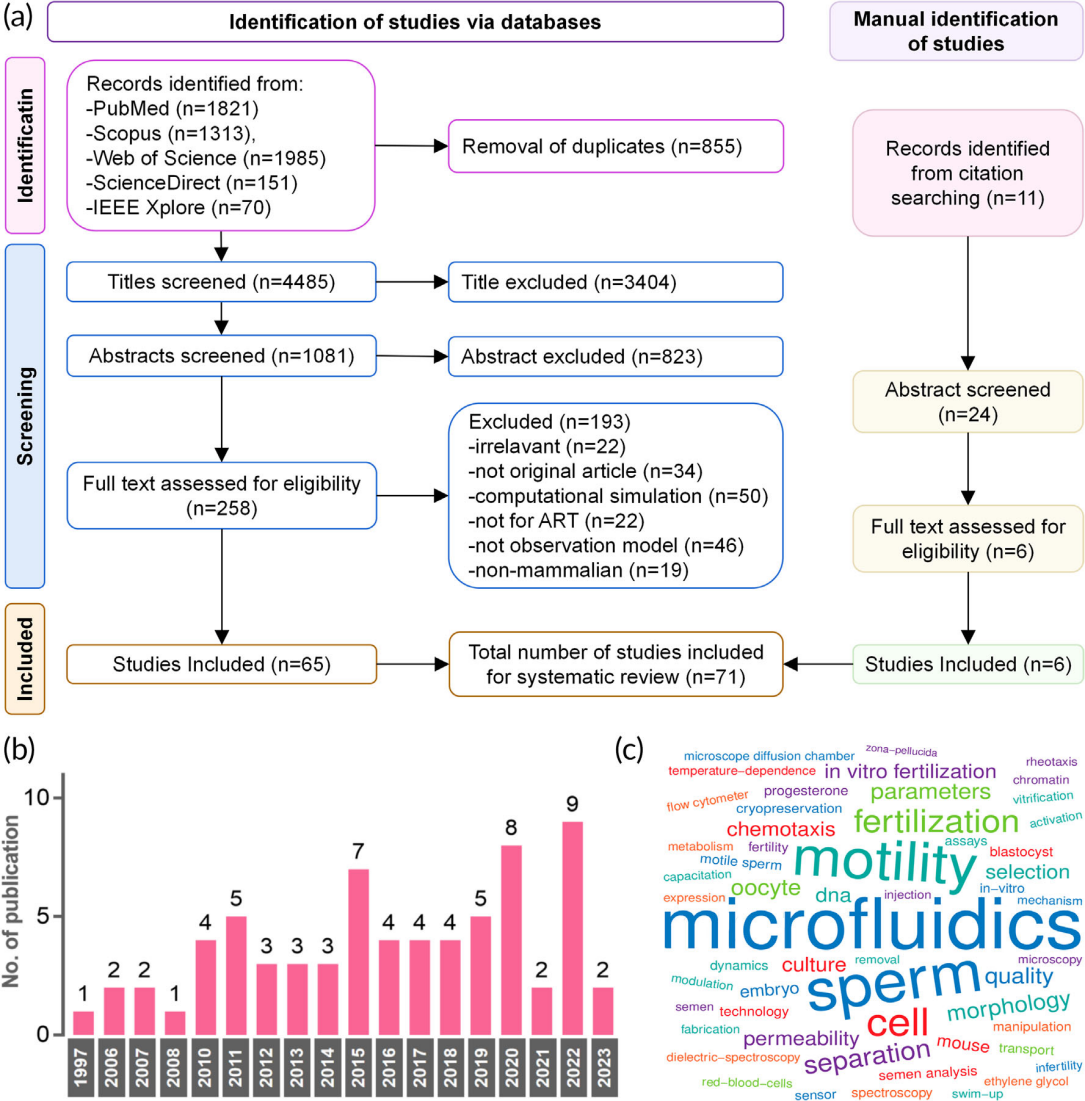
(a) PRISMA flow diagram. (b) Histogram of eligible publications by year. (c) The 55 most used keywords to reflect diverse topics in microfluidics.

The initial electronic database search resulted in 5340 papers, of which 4485 remained after removing duplicates. We excluded 3404 and 823 papers after screening the titles and abstracts, respectively. The full texts of the remaining 258 studies were assessed, excluding 193 that did not meet the inclusion criteria and retained 65 for analysis. An additional six studies were retrieved by manually searching the reference lists of the included studies. Finally, 71 articles were included in this systematic review. A flowchart of the search process is shown in Figure 2a.

### 3.2 Study characteristics

As expected, the number of papers showed a slight increasing trend from 1997 to 2023 (Figure 2b). The word cloud revealed the most relevant keywords: microfluidics, sperm, motility, fertilization, separation, and chemotaxis (Figure 2c). Equal percentages of studies used human and mouse samples (31.5%), followed by bovine (19.2%) and porcine (16.4%). The prominent country in terms of publication number was the USA (*n* = 13), followed by China (*n* = 12), the Netherlands (*n* = 7), and Japan (*n* = 7). As for institutions, seven studies were reported by researchers from the University of Twente, followed by four studies each from Assuit University and the University of Science and Technology of China. Polydimethylsiloxane (PDMS; *n* = 49) was the most used material, followed by glass (*n* = 9), silicon (*n* = 4), and polymethyl methacrylate (PMMA; *n* = 3). The top fabrication methods were soft lithography (*n* = 28), photolithography (*n* = 9), etching (*n* = 8), and pour molding (*n* = 3).

### 3.3 Sperm examination

**FIGURE 3:**
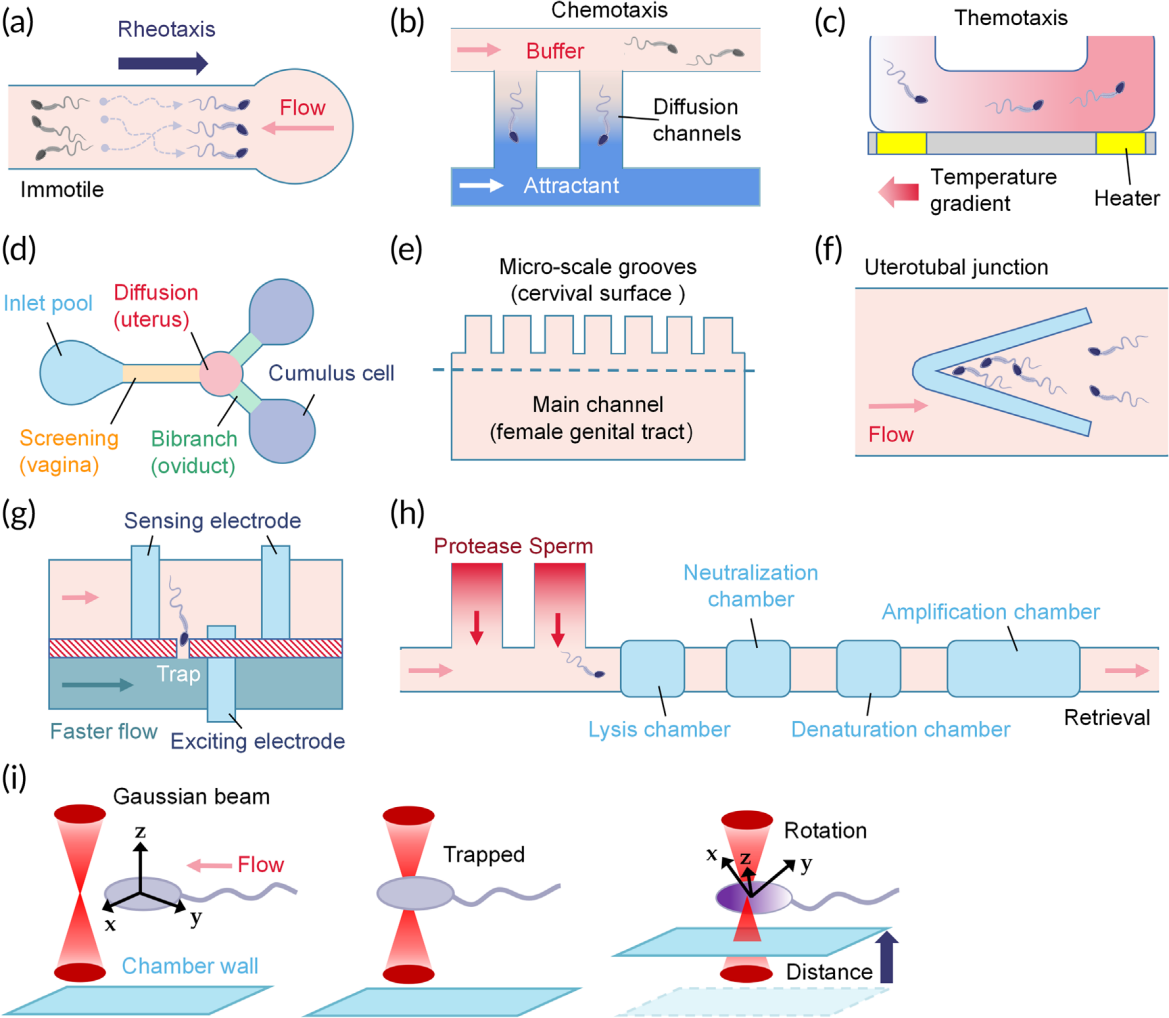
Examples of microfluidic strategies for sperm assessment. (a) The most used method to test for positive sperm rheotaxis. The immotile/dead spermatozoa (gray) are washed out with the flow, while the motile ones swim through the flow. (b) Generation of a uniform concentration gradient for chemotaxis assays. (c) A glass substrate with implemented microheaters to induce thermotaxis.^55^ (d) A chemotactic structure that simulates the entire female genital tract.^34^ (e) A microfluidic device that mimics the main reproductive tract components (bottom), including the micro‐scale grooves in the cervical surface (upper).^39^ (f) Probes with angles of 20°, 30°, and 50° were fabricated to form a uterotubal junction and trap sperm.^51^ (g) The Spermometer consists of two main channels. The spermatozoa are trapped in the interconnecting channel because of the higher fluid flow in the bottom channel. The electrode arrays are used for differential impedance analysis.^65^ (h) The microfluidic device was designed to perform single sperm whole‐genome amplification.^62^ (i) The spermatozoa are optically trapped by the beam waist of a highly focused Gaussian beam (left and middle).^68^ The spermatozoa undergo rotation and switchback oscillation when the laser beam waist is closer to the chamber wall.

The percentage of infertility attributable to male factors worldwide cannot be neglected, with rates of 60%–70% in the Middle East and 56% in Central/Eastern Europe.^28^, ^29^ Defective spermatozoa were associated with abortions, premature births, and metabolic and neurodevelopmental disorders in the offspring.^30^, ^31^ Sperm examination involves morphology, dynamic mechanics, and quantity. Of the 38 studies assessing sperm function, 15 assessed human sperm, the earliest of which was from 1997 (Table 1). Twenty‐six studies focused on sperm motility (Figure 3a–c).^20^, ^32^, ^33^, ^34^, ^35^, ^36^, ^37^, ^38^, ^39^, ^40^, ^41^, ^42^, ^43^, ^44^, ^45^, ^46^, ^47^, ^48^, ^49^, ^50^, ^51^, ^52^, ^53^, ^54^, ^55^, ^56^ Among them, five tested sperm chemotaxis ability using ovarian fragments,^32^ oviductal fluid,^54^ cumulus cells,^34^ acetylcholine,^55^ or progesterone.^45^ Four studies mimicked the female reproductive tract when assessing sperm motility, including the ovary,^34^ cervix,^39^, ^46^ and fallopian tube,^51^ aiming to simulate the natural in vivo conditions (Figure 3d–f). Five studies assessed sperm numbers.^57^, ^58^, ^59^, ^60^, ^61^ Among the remaining seven studies,^62^, ^63^, ^64^, ^65^, ^66^, ^67^ one studied impedance^65^ (Figure 3g), one applied whole‐genome analysis (Figure 3h),^62^ one studied viability,^63^ one studied beat frequency,^64^ one studied opacity,^67^ one perform digital holography,^68^ and one integrated interferometric phase microscopy.^66^

**TABLE 1:** Main observations of microfluidics‐based sensors in assisted reproductive technology.

	Reference	Species	Group	Main finding
Sperm				
Motility	Kricka et al. (1997)	Human	① Makler chambers ② Microfluidic chips	A good correlation of the sperm motility testing in ①②
	Koyama et al. (2006)	Mouse	Chemotactic and buffer chambers within chips	More sperm swim toward the ovarian extract.
	Ohta et al. (2010)	Human	Not applicable	Distinguishing motile and non‐motile sperm using optoelectronic tweezers with little DNA damage.
	Xie et al. (2010)	Mouse	Chips with various lengths and widths	Higher percentage of motile sperm by a straight channel 7 mm in length and 1 mm in width.
	Matsuura et al. (2011)	Porcine	① Glass substrate ② PDMS substrate	Significant decreases in the linear velocity and amplitude of lateral head displacement in ① than ②.
	Chen, Chen et al. (2013)	Human	No applicable	Characterization of straight‐line velocity and beat frequency in a microfluidic device.
	El‐Sherry et al. (2014)	Bovine	Various flow velocities and shear stresses in chips	Confirmation of the positive rheotaxis and wall tracking behavior.
	Tung et al. (2014)	Bovine	Various fluid flow and surface topography in chips	The microfluidic grooves embedded on a channel surface facilitate sperm migration against fluid flow.
	Frimat et al. (2014)	Porcine	No applicable	Sperm were trapped by micro‐contact printing with high patterning efficiency and viability, and were used for motility analysis.
	Bukatin et al. (2015)	Human	Various kinematic viscosities and shear rates in chips	Analyzing 3D rheotactic turning behaviors of sperm.
	Elsayed et al. (2015)	Bovine	① CASA plugin ② Modified plugin	Better performance and parameters in ② than ①.
	Nosrati et al. (2016)	Human	① Standard approaches ② Chips	Measuring live and motile sperm concentrations and motility quantitatively.
	Bhagwat et al. (2018)	Mouse	① Standard medium ② Gradient ACh ③ Uniform ACh	Studying sperm chemotaxis and chemokinesis using Ach.
	Ko et al. (2018)	Mouse	① Chemotaxis ② Thermotaxis ③ Chemotaxis+thermotaxis ④ Control	More sperm arrived at the outlet in ①②③ than ④.
	You et al. (2019)	Human	No applicable	A 1000‐trap microarray helps align and array individual live sperm.
	Abdel‐Ghani et al. (2020)	Sheep	① PR ≥ 40% ② PR < 40%	Higher pregnancy rate and fewer pregnancy loss in ① than ②.
	Berendsen et al. (2020)	Porcine	① No progesterone ② Progesterone	Higher chemotactic rate for spermatozoa in ② than ①.
	Yan et al. (2020)	Human	① Human tubal fluid ② Methylcellulose	A one‐step selection and evaluation of high‐quality sperm.
	Doostabadi et al. (2022)	Human	① Control ② Chemotaxis ③ Thermotaxis ④ Chemotaxis + thermotaxis	Decreased concentration, DNA fragmentation index, and higher progressive motility and acrosome reaction in ②③④ than ①.
	Pan et al. (2022)	Porcine	No applicable	The chip was coupled with a portable microscopic imaging system to evaluate sperm quality.
	Yu et al. (2022)	Human	① BWW ② Hyaluronic acid	Constructing a cervix chip filled with hyaluronic acid.
	Sharma et al. (2022)	Human	① Raw semen ② Control (no‐flow) ③ Chips	Higher percentage of morphologically normal sperm and lesser DNA fragmentation in ③ than ①②.
	Yaghoobi et al. (2022)	Bovine	No applicable	Combining RHEOtaxis quaLity indEX and motile sperm concentration to predict sperm fertility levels.
	Gai et al. (2022)	Bovine	Various widths	Identifying sperm dynamics with/without water flow.
	El‐Sherry et al. (2023)	Human	① Normal motility ② Reduced motility ③ Infertile ④ Fertile	Comparable progressive motility in ①②. Lower PR% in ③ than ④.
	Yu et al. (2023)	Mouse	① Blank chips ② Oviductal chips	Filling chips with fibronectin and progesterone gradient to mimic isthmus and ampulla.
Counting	Segerink et al. (2010)	Porcine	① Burker counting chamber ② Chips	Determining concentration of sperm through electrical impedance measurements.
	Chen, Chiang et al. (2013)	Human	① Makler Chamber ② Sperm quality analyzer ③ Chips	①③ show strong correlation.
	Kanakasabapathy et al. (2017)	Human	No applicable	A smartphone‐based diagnostic assay for semen analysis in sperm concentration and motility.
	Phiphattanaphiphop et al. (2019)	Bovine	Various impedance and detected under CASA	Correlation with *R* ^2^ = 0.9997.
	Kim et al. (2020)	Human	① Commercial chips ② Modified chips	Correlation with *R* ^2^ = 0.9618
Others	Wang et al. (2012)	Human	No applicable	A high‐throughput method for single‐cell whole‐genome analysis that was used to measure the genomic diversity.
	Merola et al. (2013)	Bovine	No applicable	Biovolume estimation of sperm cells through combining the optical tweezers technique with the digital holography.
	de Wagenaar et al. (2015)	Porcine	Various heights in microchannels	Entrapment of single sperm cell to study cell viability, chromosomal content, and acrosome state.
	de Wagenaar, Dekker et al. (2016)	Porcine	Not applicable	Simultaneous detection and sorting of morphologically normal sperm cells through droplet impedance measurement.
	de Wagenaar, Geijs et al. (2016b)	Porcine	Various temperature and chemical stimuli	Single sperm analysis of beat frequency.
	Eravuchira et al. (2018)	Human	No applicable	Real‐time automatic analysis based on the 3D morphology and contents.
	Kruit et al. (2022)	Porcine	① Control ② A23187 treated	The opacity at 19 MHz over 0.5 MHz is associated with acrosome integrity.
Oocytes				
Mechanics	Liu et al. (2010)	Mouse	Not applicable	Young and old oocytes can be distinguished through force measurement.
	Arai et al. (2015)	Bovine	Not applicable	A high‐through put cell mechanical characterization method using a robot integrated microfluidic chip
	Nakahara et al. (2015)	Mouse	Not applicable	Simultaneous transportation and mechanical measurement of oocytes through a micropillar array in an open environment.
	Luo et al. (2015)	Mouse	Not applicable	Evaluating the oocyte's spindle via a constricted microfluidic channel.
	Nakahara et al. (2018)	Mouse	Not applicable	The Young's modulus of the zona pellucida of cryopreserved oocytes increases along with the cultivation time.
	Pokrzywnicka et al. (2019)	Porcine	Not applicable	Changes in the surface areas and diameters of the deformed oocyte depend on the quality class based on COC morphology.
	Saffari et al. (2023)	Mouse	Not applicable	Oocytes' cortical tensions of very low or very high lead to unsuccessful growth.
Permeability	Zhao et al. (2017)	Human	Various CPAs and temperature	Characterization of the CPA‐ and temperature‐dependent permeability of the oocyte membrane.
	Chen et al. (2019)	Human	Various CPAs and temperature	Dividing oocytes into high‐ and poor‐quality ones according to the membrane permeability.
	Lei et al. (2019)	Mouse	Various CPAs and temperature	Testing the membrane permeability of oocytes exposed to different conditions.
	Guo et al. (2020)	Human and mouse	Various types and concentrations of CPAs	Analysis of oocyte volume.
	Chen, Memon et al. (2020)	Mouse	Various CPAs and continuous concentration change	Analysis of multiple oocytes volume simultaneously.
	Tu et al. (2022)	Bovine	① Manual estimate ② Computational technique	①② are comparable to estimating ellipsoidal and spherical volumes.
Microspectrometry structure	Zeggari et al. (2007)	Not reported	Not applicable	Quantifying the maturity degree and fertilization of oocyte to use as a fertilization indicator.
	Sniadek et al. (2011)	Porcine	Not applicable	Dividing oocytes into class 1 and class 2 using microspectrometry.
	Walczak et al. (2011)	Porcine	① Control ② Actinomycin D ③ Ethanol	Supravital examination of single embryos for the presence of apoptotic blastomers.
	Angione et al. (2015)	Human and mouse	Not applicable	Real‐time and longitudinal imaging of oocytes following fluorescent labeling.
Impedance	El Hasn et al. (2017)	Mouse	① Zona pellucida intact oocytes ② Zona pellucida‐free oocytes	Higher impedance in ② than ①.
	Azarkh et al. (2023)	Mouse	① Wide‐type ② Double knock out	Characterizing the Young's modulus of the oocyte zona pellucida.
Oxygen	Tedjo et al. (2021)	Bovine	Not applicable	Analysis of oxygen consumption rate and oxygen flux density of COCs.
Embryos				
Oxygen	O'Donovan et al. (2006)	Mouse	① Two‐cell ② Blastocyst	Characterization of oxygen consumption in ①② over a one‐hour time period.
	Wu et al. (2007)	Bovine	Not applicable	A microfluidic chip with the built‐in amperometry detector array to measure the oxygen consumption of a single embryo.
	Date et al. (2011)	Mouse	① Two‐cell ② Morula ③ Blastocyst	Increased oxygen consumption is found.
	Kurosawa et al. (2016)	Human and bovine	Various stages and culture duration	Sensing the oxygen consumption rate of spheroids, bovine embryos and frozen–thawed human embryos, and it corresponds to the developmental potential of embryos.
	Hiramoto et al. (2017)	Mouse	① Microfluidic chip ② SECM	①② are the same in detecting oxygen concentration profiles surrounding the embryos.
Mechanics	Bae et al. (2011)	Bovine	Various amplitude and duration of the stimulus	The potential of embryos is compromised after treatment.
Viability	Śniadek et al. (2012)	Mouse	① Blank ② Non‐apoptotic ③ Apoptotic	Utilizing miniaturized fluorescence to discriminate ①②③.
Secretion	Urbanski et al. (2008)	Mouse	Not applicable	Measuring glucose, pyruvate and lactate with sub‐microliter volumes within 5 min.
	Heo et al. (2012)	Mouse	Not applicable	Real time culture and analysis of embryo glucose level with a deformation‐based actuation.
	Chen, Sun et al. (2020)	Human	Not applicable	Combination of microfluidic droplets and multi‐color fluorescence to accurately detect β‐hCG secreted by single embryos.
	Lee et al. (2020)	Human	Not applicable	On‐chip immunoassay for human IL‐1β and TNF‐α.
Structure	Vandormael‐Pournin et al. (2021)	Mouse	Not applicable	An eggbox imaging device designed to image preimplantation embryos.
	Sivelli et al. (2022)	Bovine	Not applicable	Using nuclear magnetic resonance technology to observe single embryos for selection.

Abbreviations: 3D, three dimensional; Ach, acetylcholine; BWW, Biggers‐Whitten‐Whittingham; CASA, computer‐assisted sperm analysis; COC, cumulus‐oocyte complex; CPA, cryoprotectant; IL‐1β, interleukin 1β; PDMS, polydimethylsiloxane; PR, positive rheotaxis; SECM, scanning electrochemical microscopy; TNF, tumor necrosis factor; β‐hCG, human chorionic gonadotropin beta.

### 3.4 Oocyte evaluation

**FIGURE 4:**
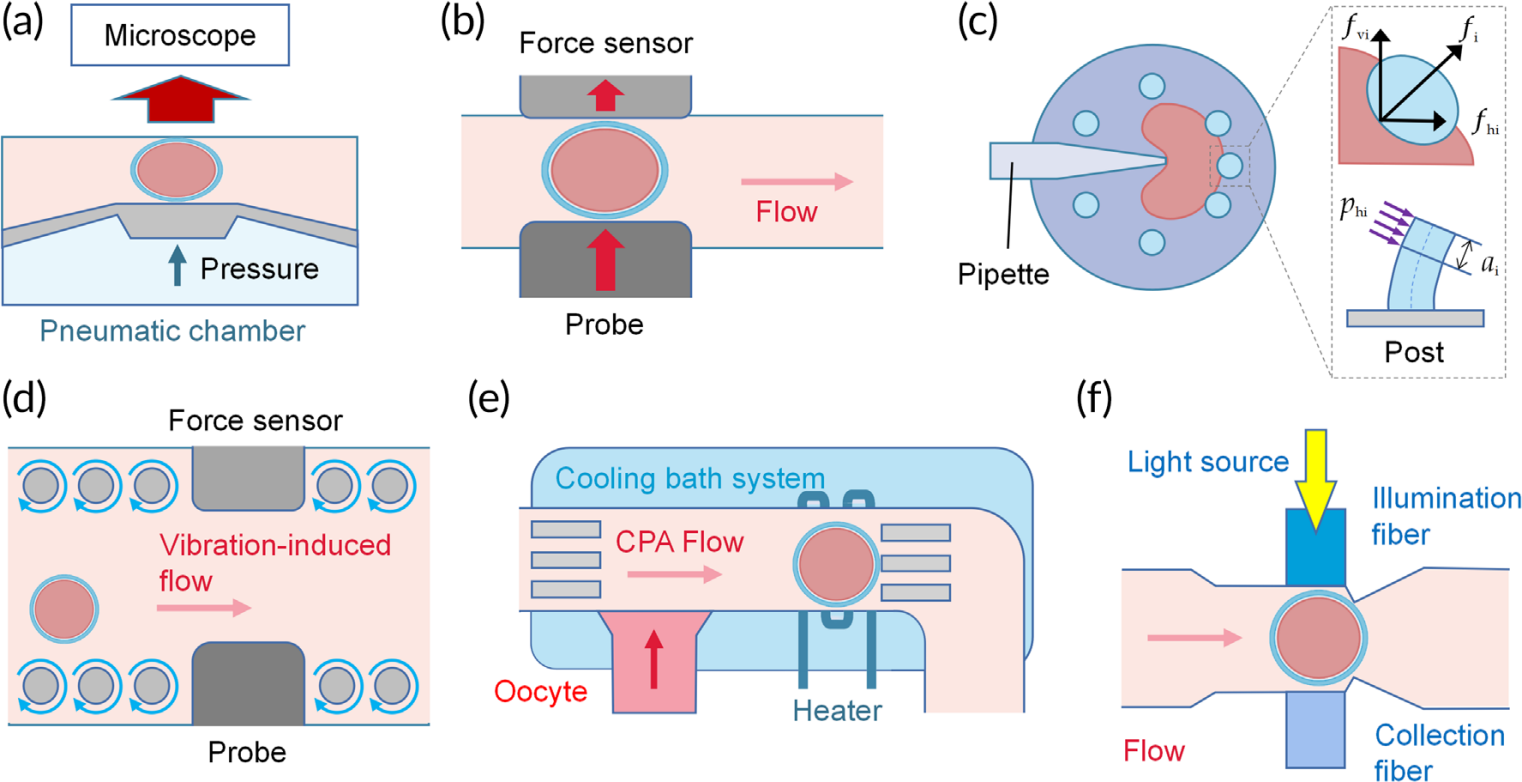
Schematic representations of microfluidic platforms for oocyte examination. (a) Visualization of a deformation microcytometer with a trapped oocyte.^74^ (b) An external force is applied to push the oocytes, and the force sensor measures the reaction force.^73^ (c) The forces deform the oocytes and deflect three supporting posts. Force balance on cells under indentation and the post deflection model (inset).^70^ (d) The mechanical characteristics of oocytes in an open environment. The vibration‐induced flow serves as a cell transport mechanism.^72^ (e) Single‐cell detection with fluorometric readout.^83^ (f) Microfluidic platforms with integrated local temperature control system and capture module.^79^

The properties of oocytes have always interested researchers and clinicians. Twenty studies characterized oocytes using microfluidics (Table 1).^56^, ^69^, ^70^, ^71^, ^72^, ^73^, ^74^, ^75^, ^76^, ^77^, ^78^, ^79^, ^80^, ^81^, ^82^, ^83^, ^84^, ^85^, ^86^, ^87^ Microfluidic chips can measure large numbers of oocytes with high throughput and sensitivity.^88^ Seven studies deformed oocytes to test their mechanical properties (Figure 4a–d),^69^, ^70^, ^71^, ^72^, ^73^, ^74^, ^75^ including one that applied injection force to test oocyte mechanics (Figure 4c),^70^ and three that used compression force. Six studies utilized microfluidic tools to investigate cellular membrane permeability to cryoprotectants (CPAs; Figure 4e),^56^, ^76^, ^77^, ^78^, ^79^, ^80^ which are associated with cryopreservation efficacy. Two of the four microspectrometry studies classified the oocytes into top‐ and low‐quality (Figure 4f).^82^, ^83^ Less frequently reported properties included current impedance (*n* = 2)^85^, ^86^ and oxygen concentration (*n* = 1).^87^

### 3.5 Embryo evaluation

**FIGURE 5:**
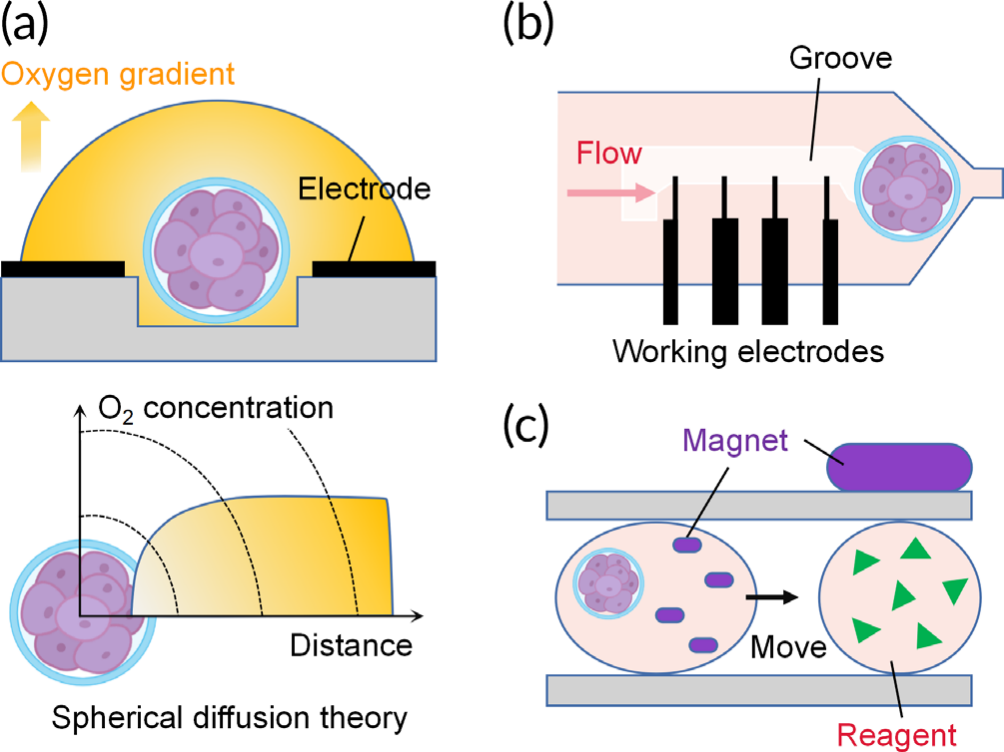
Schematic representations of microfluidic platforms for embryo analysis. (a) The concentration gradient of dissolved oxygen is formed by embryo respiration in a hemispherical area (top). The explanation for the spherical diffusion theory (bottom).^89^, ^90^ (b) Layout of electrochemical electrodes to estimate in situ oxygen consumption.^93^ (c) A droplet containing embryos and magnetic beads merges with a reagent solution under magnetic force.^98^

The advantages offered by microfluidics are well suited for studying embryogenesis. Much progress has been made in simulating the in vivo microenvironment to improve mammalian embryo development. However, advancements in assessing their function are limited. Thirteen studies evaluated embryos.^89^, ^90^, ^91^, ^92^, ^93^, ^94^, ^95^, ^96^, ^97^, ^98^, ^99^, ^100^, ^101^ Oxygen consumption was investigated by five groups (Figure 5a,b),^89^, ^90^, ^91^, ^92^, ^93^ while only one study assessed embryo mechanics.^94^ Reliable detection of soluble factors at the single‐embryo level presents great potential but is challenging. Human chorionic gonadotropin beta secretion was measured by detecting an enzyme‐induced fluorescence marker with a lower sensitivity limit of 0.1 pg/mL (Figure 5c).^96^ Real‐time analysis of glucose metabolism was assessed at the single‐embryo level over 24 h.^97^ Simultaneous detection of tumor necrosis factor and interleukin 1β was achieved using digital microfluidics.^98^ Triple metabolite (glucose, pyruvate, and lactate) detection was achieved within 5 min by serial measurements.^99^ Cell apoptosis was investigated in one study that assessed the viability.^95^ Two studies evaluated embryo structural alterations.^100^, ^101^

## 4 DISCUSSION

Mounting evidence supports the notion that sperm/oocyte quality profoundly affects fertilization, early embryonic survival, pregnancy maintenance, and fetal development.^102^, ^103^ Various microfluidic systems have been used in reproductive biology to drive ongoing research on developmental competence assessment. However, limited knowledge was transferred from laboratories to clinics. The field still lacks a comprehensive understanding of the up‐to‐date assessment of microfluidic applications in ART. This systematic review summarized the current state of microfluidics in detecting the motility, mechanics, structure, and oxygen consumption of gametes and embryos, shedding light on novel perspectives for future ART development.

### 4.1 Principal findings

#### 4.1.1 Sperm motility patterns controlled by microfluidics technology

Male factors are present in half of infertility cases and are affected by cultural, environmental, genetic, and age factors.^104^, ^105^ Conventional semen analysis includes the volume, concentration, motility, and morphology. Microfluidics offers many benefits for sperm examination, including stable concentration/temperature gradients, controllable flow direction, and shear rate. With the help of microfluidic devices, additional information about chromosomal content, acrosome integrity, and genomic alteration could be collected to evaluate the reproductive potential of males. Furthermore, sperm manipulation using microfluidics is advantageous as it can introduce automation and scalability and causes less DNA damage than standard techniques.^106^ Therefore, investigating the application of microfluidics is essential for sperm observation and selection during ART.

**FIGURE 6:**
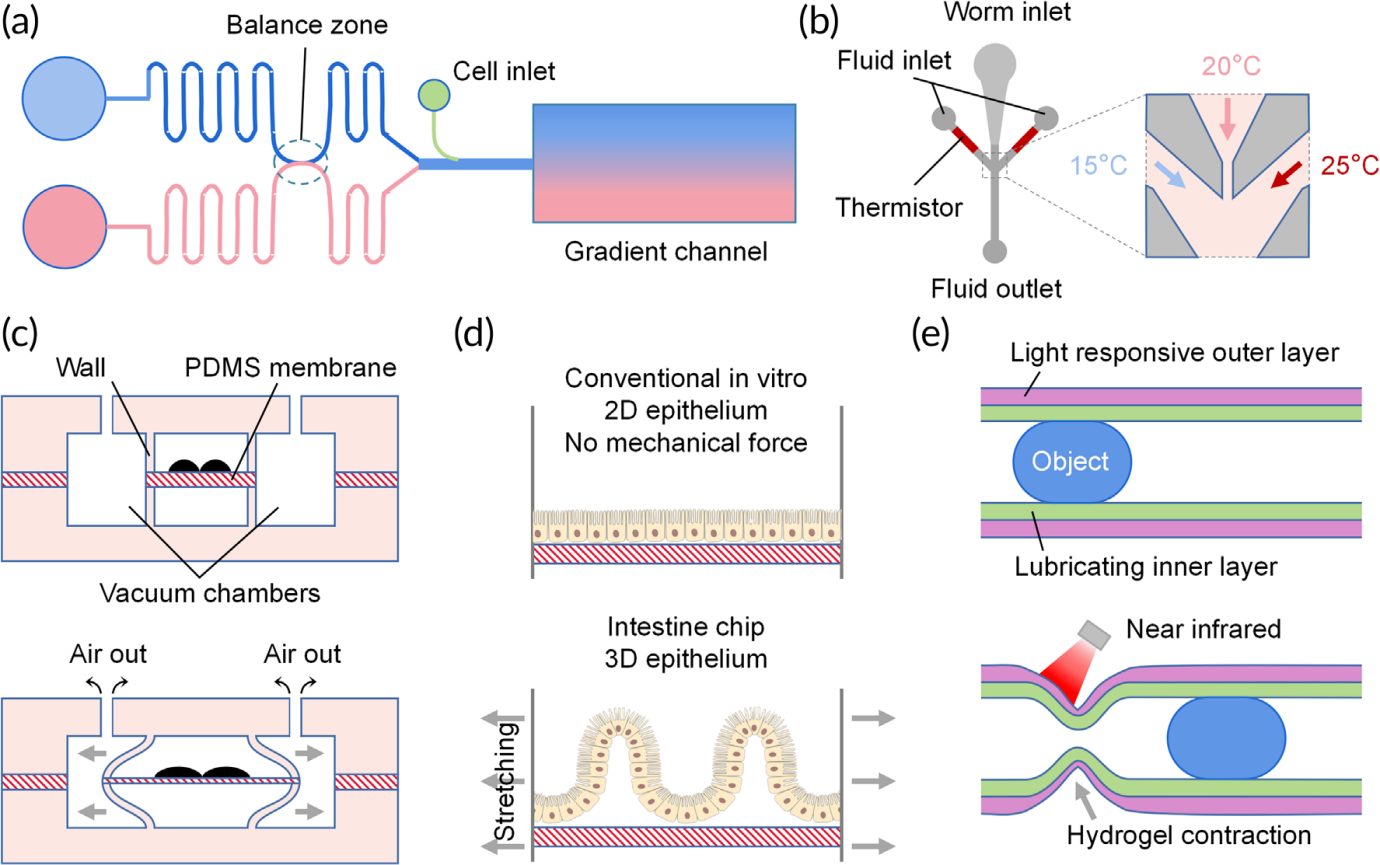
Microfluidic platforms in other applications. (a) An illustration of a microfluidic gradient generator with a pressure balance zone. (b) A schematic of the thermotaxis assay chip for nematodes. The arrows represent the flow directions in the fluid and vacuum channels.^108^ (c) An illustration of the lung‐on‐a‐chip working principle.^109^, ^110^ (d) 3D villus‐like structures in a gut‐on‐a‐chip. Lateral arrows indicate lateral stretching by the side chambers.^111^ (e) A schematic showing an artificial tubular hydrogel actuator structure and the motion of an object driven by an applied near‐infrared light.^112^

Mucus secretion, ciliary movement, and muscle contractions within the female reproductive tract create fluid flow opposite to the sperm swimming direction. To arrive at the site of fertilization, spermatozoa must enact rheotaxis, chemotaxis, and thermotaxis. Rheotaxis is the sperm swimming against the flow direction (Figure 3a). The rheotaxis level positively correlates with advanced chromatin maturity, well‐organized morphology, higher motility, and reduced DNA fragmentation.^51^ Chemotaxis refers to organism migration along a concentration gradient (Figure 3b). A decreasing concentration gradient is formed in the diffusion channels from the attractant chamber to the buffer chamber.^54^ In response to chemicals, spermatozoa switch their swimming directions toward the higher concentration. Sometimes, a balance zone can be added to stabilize the flow pressure from various inlets and lower the operation accuracy requirement (Figure 6a).

Thermotaxis describes the movement of spermatozoa along temperature gradients, as the oviduct temperature is 1–2°C higher than the uterus in rabbits and pigs (Figure 3c).^51^, ^107^ Microheaters are placed beneath the inlet and outlet to produce a linear temperature gradient in the microchannels, enabling the study of thermotaxis behavior (Figure 3c). Some studies simultaneously tested the response to various temperatures by fabricating more complex microfluidic chips, a task achieved with nematodes (Figure 6b).^108^ The arms of Y‐shaped microchannels were embedded with thermistors to produce stable temperatures. The thermotaxis behavior was validated by cultivating the nematodes at 20°C and exposing them to streams of 15°C and 25°C. The coexistence of thermotaxis and chemotaxis better represents the in vivo environment than single factor conditions^55^ and should be more accepted by researchers.

Interestingly, mechanical deformations such as uterine peristalsis could also affect sperm motility. However, no such microfluidic chips have been reported, possibly due to the limited progress in this field. Developing deformable devices will greatly inspire innovative designs for studying sperm motility. For example, in the mechanically active lung‐on‐a‐chip, a vacuum is applied in the side chambers to deform the thin walls and flexible membrane, so as to replicate dynamic distortion of the alveolar‐capillary interface caused by the breathing movements (Figure 6c).^109^, ^110^ Following a similar approach, the human intestinal epithelium spontaneously forms 3D villus‐like structures (Figure 6d).^111^ Zhang et al. developed tubular intestine‐like actuators that contract after exposure to near‐infrared light. Super‐hydrophilic alginate gels were added to the inner layer to mimic the gastrointestinal peristalsis (Figure 6e).^112^ This design could be useful when fabricating artificial fallopian tubes, whose inner surface has cyclic peristaltic contraction ability and is covered by a mucous layer. Taken together, the microfluidics technology has been widely implemented to investigate sperm motility. Promising results could be obtained by designing more creative chips.

#### 4.1.2 Synchronous imitation of female reproductive tract microenvironment

The female reproductive tract plays an important role in interacting with the spermatozoa through complex secretory fluid and epithelial cells lining the lumen.^113^ Fabrication of an in vitro platform that mimics the microenvironment of the female reproductive tract could enable a more careful examination of the spermatozoa, as their status will be close to that in vivo.

Many components in the cervical canal, including mucin, cytokines, defensins, and cervical mechanics, could be considered when fabricating cervix chips to assess sperm motility. Cervical mucus is composed of 92%–95% water and ions and 5%–8% solid matter.^114^ However, it cannot be recapitulated by conventional cervicovaginal culture models.^115^ With the help of the microfluidic technique, Izadifar et al. successfully developed a human cervix chip that could produce abundant amounts of mucus, mimicking the natural one biomechanically and biochemically.^115^ A cervix chip could also be fabricated by culturing cervical epithelial cells on the mucus chips.^116^ To date, only hyaluronic acid and methylcellulose have been added to construct cervix chips,^46^, ^49^ while co‐culturing sperm and cervical cells within one microfluidic chip is yet to be reported. Defensins, mainly antibacterial and antiviral peptides, are immunomodulatory substances detected in the female reproductive tract.^117^ When spermatozoa pass through the cervical canal, defensins are loaded onto their surface and promote transportation, acrosome reaction, and fertilization. As a growing body of evidence indicates the importance of defensins to fertility, their function could be studied on sperm that are cultured microfluidic chips. Moreover, since the cervix is well known for its mechanical changes during pregnancy, a cervix chip coated with type I and III collagens and smooth muscle cells might be a feasible way to recapitulate organ contractions.^118^

Structural interactions in the uterus, utero‐tubal junction, and fallopian tubes with the spermatozoa are essential to understanding factors that determine male and female fertility. Xie et al. designed a motility screening channel (cervix), diffusion chamber (uterus), and bi‐branch channels (oviducts) in one microfluidic chip and added cumulus cells as the chemoattractive source to investigate sperm motility (Figure 3d).^34^ Microgrooves are surface topography structures found in the bovine cervical canal. To recreate this, 20‐μm wide and 20‐μm deep microchannels were designed (Figure 3e). Furthermore, probes with angles of 20°, 30°, and 50° were embedded in a microfluidic platform to recapitulate the utero‐tubal junction (Figure 3f).^51^ Sperm with velocities less than the rheotaxis velocity and debris are washed away and sperm with higher velocities are collected in the probe. Microfluidic experimental tools that model the complex biophysical female reproductive tract will have direct applications in designing efficient fertilization systems and studied the mechanism of motile sperm.

#### 4.1.3 Integrated microfluidic and advanced equipment

One of the advantages of microfluidics is the ability to couple devices with machine‐learning algorithms, real‐time cameras, and electromagnetic devices, making them more powerful tools.^40^ Computer‐assisted sperm analysis (CASA) is a popular and important method to assess sperm quality using machine learning and microscopic cinematography. Through the processing of a large number of images, the curved‐line velocity, straight‐line velocity, averaged‐path velocity, beat cross frequency, and other parameters are calculated to reflect the semen quality.^119^ Some open‐access plugins based on ImageJ software, such as CASA_automated and CASA‐bgm, can also analyze sperm motility and are suitable especially for ART centers that cannot afford expensive commercial software.^120^, ^121^ To improve CASA accuracy, Elsayed et al. incorporated wall‐tracking behavior and rheotaxis into the analysis of sperm cultured in microfluidic chips.^41^ Besides, capturing the 3D helical motion of sperm was reported to benefit sperm quality judgment.^122^

Most sperm quality‐based studies relied on population‐based approaches, making them unsuitable for obtaining single‐cell information. In a report by Wagenaar et al., individual sperm was trapped under a pressure gradient induced by differential flow rates (Figure 3g).^63^ The viability, chromosome content, and acrosome status of each entrapped cell were assessed. Wang et al. developed a single‐spermatozoon whole‐genome analysis method to gain insights into genetic changes among cells (Figure 3h).^62^ During the procedure, protease is first introduced to treat single sperm and generate chromosome suspension in the lysis chamber. Then the suspension is neutralized and subjected to an amplification reaction. Finally, the amplification products are collected for downstream analysis. This technique was shown to achieve high‐fidelity single‐chromosome amplification.

The incorporation of smartphone‐based platforms with microfluidic chambers is convenient for point‐of‐care testing. These devices have been used to assess sperm viability, DNA fragmentation, motility, and hyaluronic acid binding. The technology could also help make sperm testing available for socioeconomically challenged communities worldwide.

#### 4.1.4 Cellular mechanics related to the reproductive performance of oocytes and embryos

The stiffness and deformation characteristics of single cells can be used as label‐free determinants of biological status. The cortical tension of oocytes drops six‐fold during oocyte maturation from prophase I to metaphase II and then increases two‐fold upon fertilization.^123^ These mechanical alterations are related to the dynamics of the actin, myosin‐II, and Ezrin/radixin/moesin families. Intracellular mechanics, including spindle position and polar body extrusion, also participate in oocyte meiosis.^124^ The ZP is a thick extracellular matrix that coats mammalian oocytes and early‐stage embryos. Its viscosity and elasticity increase approximately four‐ and three‐fold, respectively, at fertilization.^125^ Furthermore, studies have identified increased tension in zygotes at the beginning of embryonic cleavage.^126^, ^127^ Embryos could even be injected with nanodevices to reflect cytoplasmic mechanical activity and investigate the intracellular forces.^128^ Cellular mechanics is closely related to fertility capacity, so it could serve as a credible indicator of reproductive performance.

Modern microfluidic devices enable cellular mechanics characterization to assess cellular activity. Aleksandra et al. investigated oocyte deformability by measuring changes in diameter and surface area under 100 kPa compression (Figure 4a).^74^ They found that changes in the ooplasm surface area amplitude were positively correlated with decreasing quality and suggested that the fluctuations might be due to the gap between the ooplasm and the ZP.^74^ In another study, a micropipette was controlled to indent individual oocytes held in an elastic device (Figure 4c).^70^ Force data were collected from the supporting posts during the indentation process. However, the micropipette diameter was considerably smaller than the oocyte diameter, so the applied force might not have affected the cell evenly. Another group measured the mechanical characteristics of oocytes in a robot‐integrated microfluidic chip (Figure 4b).^72^, ^73^ A mechanical probe pushed oocytes in the measuring position toward the force sensor. The manipulation force and resolution were in the nanonewton and angstrom orders, respectively. They further fabricated open‐environment microfluidic chips, driven by vibration‐induced flow, to prevent bubble formation when moving the probe and sensor (Figure 4d). Collectively, the findings of these studies demonstrate the critical roles the mechanical properties of oocytes play. Microfluidic chips are anticipated to open a window into mechanical assessment and provide information on female reproduction cellular mechanics.

#### 4.1.5 Morphological and optical parameters to predict gamete and embryo quality

Studies on applying microfluidics to evaluate single sperm morphology are limited. According to the World Health Organization (WHO) manual 5th edition strict morphology criteria, the average normal sperm morphology rate is 4%.^129^ The sperm head, midpiece, and tail parameters are elaborately defined in the manual. For example, a normal head is defined as having an oval shape with smooth contours and partially covered by the clearly visible, well‐defined, and homogenous stained acrosome.^130^ However, recent evidence showed that sperm morphology was poorly associated with ART and natural pregnancy outcomes, presumably accounting for the rare use of microfluidics to assess morphological indexes.

Traditional morphological evaluations of oocytes and embryos are based on scoring systems and classifications of polar bodies and spindles.^131^, ^132^ These systems provide valuable information for selecting high‐quality oocytes and maximizing embryonic developmental outcomes. However, they are also controversial because they are subjective and inaccurate. Recently, optical measurements have been proposed to assess oocyte maturity and fertilization status. Perfectly aligned illumination and collection fibers were set in microfluidic chips, with the former connected to a light source and the latter to a spectrometer (Figure 4f).^84^ The light propagated through the oocyte during the measurement, and the transmission spectra were recorded and processed for further analysis. It was found that shorter wavelengths but higher arbitrary units were recorded in fertilized oocytes than in matured ones.^84^ This system simplifies the oocyte manipulation process and is less invasive than conventional methods. Similarly, the quality of oocytes could be measured by a microspectrometric visible/near‐infrared system. It was shown that numerical shifts of the maximum light transmission near 580 and 620 nm could be used to determine oocyte quality in cows and pigs, respectively.^133^

It is well‐recognized that apoptosis plays an essential role in oocytes and embryos. It causes the elimination of over 99% of the germ cells through follicular atresia.^134^ The initiation of the apoptotic cascade in early embryos was associated with implantation failure, cleavage, and developmental incompetence.^135^ Common apoptotic staining methods include detecting relevant proteins, terminal deoxynucleotidyl transferase dUTP nick end labeling, and annexin V or propidium iodide. Image acquisition is traditionally achieved by placing microfluidic channels in standard bench‐top optical microscopes. However, some reagents and dyes are toxic and mutagenic, precluding further culture of the assessed gametes or embryos.^136^ Walczak et al. examined the apoptotic blastomeres of single supravital embryos by integrating microfluidic chips with a fluorescence excitation module^95^ while retaining the developmental capacity of the stained samples. The integrated optical elements or lens‐less imaging methods allow incorporating optical imaging into lab‐on‐chip systems, increasing automation, compactness, and portability.^18^

#### 4.1.6 Oxygen consumption indicator for ART evaluation

Metabolic indicators can be used to reflect embryo quality. A higher respiration rate is found in in vitro‐produced embryos than in in vivo‐produced ones, possibly due to differences in the embryo stage and distinct culture conditions.^137^ High‐quality embryos possess numerous matured mitochondria with expanded cristae, while poor‐quality ones have more lysosome‐like structures.^138^ More importantly, embryonic respiration rate was positively associated with viability following embryo transfer.^139^ Therefore, metabolism and oxygen consumption could serve as objective and quantitative parameters to evaluate embryos.

Microfluidic chips with built‐in detectors of embryonic metabolic state have been constructed. Based on the spherical diffusion theory, Date et al. implemented an electrode array in a centrally positioned microwell and demonstrated the increase in oxygen consumption from morula to blastocyst (Figure 5a).^89^ This approach is accurate, practical, and comparable to the conventional scanning electrochemical microscopy method. Wu et al. integrated microfluidic chips with electrochemical sensors to study oxygen consumption in single bovine embryos (Figure 5b).^93^ In that study, data from four aligned working electrodes were obtained to build a computational model; however, the embryo radius had to be measured. More recently, a chip‐sensing embryo respiration monitoring system has been developed, enabling automatic measurement within 1 min. This microfluidic chip version is user‐friendly, as all the operator needs to do is load the samples into the center of the platform.^91^ While much progress has been made in expanding our knowledge about the connection between metabolism and germ cells, microfluidic devices could be critically important in improving our understanding of quality evaluation.

#### 4.1.7 Integration of artificial intelligence in microfluidics

It is generally acknowledged that even after critical embryo evaluations using time‐lapse microscopic photography (TLP) and preimplantation genetic testing, the implantation rate remains difficult to predict.^140^ It might be because traditional manual assessments rely heavily on the embryologist's precision, experience, and ability.^141^ In contrast, artificial intelligence (AI) makes predictions based on complex pattern recognition by incorporating the processing power of computers.^142^ It is flexible, and the analytical principles can be modified to generate innovations when exposed to additional data, making it superior to conventional simple statistical models. Since the final decision made by AI is reached with minimal human intervention, intra‐ and inter‐operator variabilities are diminished.

AI techniques have been utilized to attain high‐quality gametes and embryos in ART laboratories. Bukatin et al. devised a mathematical model that could reproduce the experimentally observed 2D trajectory statistics and translate the 2D intensity information of motile spermatozoa into 3D positional data.^40^ The reconstruction of 3D beat patterns showed that flagellar propulsion followed a stepwise‐rotating plane, updating the previous helical models. RHEOLEX is a novel parameter that reflects sperm concentration and kinetics. It is calculated based on CASA capture and image processing methods. While no significant correlation was found between conventional motility parameters and in vivo fertility, RHEOLEX stands out for correlating well with sperm concentration, DNA fragmentation index, and in vivo fertility.^51^ The motility and survival rate of spermatozoa in a microfluidic chip could be assessed by performing multi‐target matching and utilizing a recognition algorithm, tracking algorithm, and YOLO4.^49^ In another study, 3D sperm images were obtained by combining optical tweezers with a digital hologram (Figure 3i).^68^ During the procedure, a Gaussian beam traps the spermatozoa inside the microchannels. The sperm rotation is achieved by regulating the light power and distance to the channel walls so that 2D quantitative sperm images at various angles are obtained. Subsequently, the Shape from Silhouette algorithm is used for 3D reconstruction. This work provided a contactless and quick method to rotate motile cells with a simple laser beam.

AI can assist embryologists in their daily activities and help patients achieve their goal of having a healthy baby.^143^ Sperm can be classified into five distinctive types (progressive, intermediate, hyperactivated, slow and weakly motile) by a support vector machine‐based decision tree with 89.9% accuracy.^144^ When implemented into a smartphone, AI could help assess sperm quality at home with an accuracy of 88.5%, without the need for professional technicians.^145^ An artificial neural network has been used to analyze TLP images of cytoplasmic movements to predict the competency of mouse oocytes, with an accuracy of 91%.^146^ Detection of the extruded polar body using machine learning allows for developmental stage definition and timely ICSI preparation. Current hotspots of AI applications in embryo management can be categorized into the following aspects: automatic annotation of embryo development, embryo grading, and embryo selection for implantation.^140^ In a retrospective study, data from 8886 embryos were collected to train an AI model named The Life Whisperer, which demonstrated 24.7%–42.0% higher accuracy over embryologists.^147^ Still, applying AI systems and microfluidic technology to analyze the cell stage and corresponding division intervals presents a challenge.

#### 4.1.8 Materials and methods for microfluidics fabrication

The durability, transparency, and biocompatibility of raw materials must be considered when fabricating microfluidic chips. Inorganic materials such as silicon and glass have been widely used in biology, but not for cell culture, as they do not allow air to permeate.^148^ Moreover, sperm might adsorb to the glass substrate, and their motility would be affected.^72^, ^149^ Commonly used organic materials include PDMS, PMMA, polystyrene, polyvinyl chloride, and polymethyl. PDMS offers several unique and attractive features: (i) non‐toxic, affordable, and available from commercial sources; (ii) optically transparent down to about 300 nm; (iii) intrinsically hydrophobic, but its surface can be turned hydrophilic; (iv) permeable to air to support long‐term culture. However, PDMS might assimilate hydrophobic molecules and lead to reduced drug concentration and pharmacological activity. To overcome this issue, polyurethane elastomer was proposed as it is resistant to the absorption of small hydrophobic molecules. Another material with a wide range of applications is PMMA. It is an amorphous thermoplastic with considerably better solvent compatibility than PDMS and no small‐molecule absorption. The PMMA chips are molded in one step and are inexpensive, making them suitable for point‐of‐care semen analysis.^46^, ^59^

Depending on material types and applications, the most widely used fabrication techniques include photolithography, soft lithography, injection molding, and laser ablation. Photolithography has a high wafer production capacity and is suitable for microscale manufacturing. Nevertheless, its application is restricted as it must be conducted in a clean room, requiring researchers to undergo extensive training before accessing the facility.^150^ Furthermore, photolithography allows little or no control over surface chemistry and is inapplicable to curved or non‐planar substrates. The alternative, soft lithography, is the technology most research groups adopted. The technique is advantageous as it provides a rapid and robust prototyping approach with diverse pattern techniques, primarily printing, molding, and embossing. The procedures can be conducted in an ordinary chemical laboratory. However, the cost of the manufacturing machines is high, making the technology non‐conducive to preparing large quantities of microfluidic devices. Etching continues to act as the dominant technology in microfluidic chip manufacturing, particularly for chips with stringent alignment requirements, such as the optical fibers used to assess oocytes.^83^, ^84^ The 3D printing technology has also been used for microfluidic chip fabrication as it is low‐cost and environmentally friendly.^45^, ^151^ During the printing process, the inlet and outlet are fabricated directly by the mold, reducing the cost of drilling and eliminating the possibility of crack formation. ART can choose among many materials and methods, depending on the equipment, experience, objective, etc.

### 4.2 Highlights of designing microfluidic chips for ARTs

Several aspects must be considered when designing microfluidic devices for ARTs. Since mammalian oocytes (50–150 μm) and follicles are much larger than ordinary cells (10–40 μm), the size of microchannels and chambers should be planned accordingly.^74^ The width and height of microchannels are usually larger than 100 μm. Human and experimental animal gametes and embryos exhibit various morphological features, biophysical properties, and activity patterns in vivo. Therefore, some modifications might be needed when transferring to clinical practice. Most importantly, oocytes and embryos are valuable, necessitating such samples to be suitable for transfer after examination. Therefore, the assessment method should cause no damage to biological samples.^152^

Microfluidics has been extensively used for separating, sorting, and culture. It would be fascinating if a fully functional microfluidic chip that can assess the viability of gametes/embryos and achieve automatic manipulation and drug screening could be created. Moreover, the fabrication of microfluidic chips for sperm motility assessment could benefit from studying bacteria, paramecium, and nematodes, as chemotaxis and thermotaxis are common spatial orientation behaviors in single‐cell microorganisms as in humans.^108^ The findings of such studies are often the innovative starting point for designing microfluidic chips for sperm assessment.

## 5 LIMITATIONS

Inevitably, this study had also some limitations. First, although microfluidic chips can greatly benefit ARTs, more elaborate studies (randomized controlled trials, observational studies, and in‐house validations) using human oocytes and embryos are needed to validate their safety, efficacy, and reproducibility. Second, the lack of microfluidics popularization is a problem for its use. Some integrated systems require specialized microscopes, electrodes, and driving pumps, making them costly. Additionally, such microfluidic chips might not be universally accepted by doctors, who must be trained before using them clinically. Finally, most early studies concerning embryos were based on multiple transfers. Whether their findings could be applied to single embryo transfer should be critically evaluated. The selection strategies, scoring strategies, and algorithms should be modified to satisfy various conditions.

## 6 CONCLUSION

Microfluidics is a young but established field that holds significant potential for ARTs. It promotes more efficient, accurate, and objective sperm, oocyte, and embryo evaluations than the traditional approaches. Besides, artificial intelligence could be combined with microfluidics to assist in the daily activities of embryologists. More well‐designed clinical studies and affordable integrated microfluidic chips are needed to validate their safety, efficacy, and reproducibility.

## AUTHOR CONTRIBUTIONS

**Tong Wu:** Conceptualization (lead); funding acquisition (equal); methodology (lead); writing – original draft (lead); writing – review and editing (lead). **Yangyang Wu:** Conceptualization (equal); methodology (equal). **Jinfeng Yan:** Visualization (equal); writing – original draft (equal). **Jinjin Zhang:** Supervision (equal); writing – original draft (equal); writing – review and editing (equal). **Shixuan Wang:** Funding acquisition (equal); project administration (lead); supervision (equal).

## FUNDING INFORMATION

This work was financially supported by the grants from the National Key Research and Development Program of China (No. 2022YFC2704100) and National Natural Science Foundation of China (No. 82301849).

## CONFLICT OF INTEREST STATEMENT

The authors declare that they have no known competing financial interests or personal relationships that could have appeared to influence the work reported in this paper.

### PEER REVIEW

The peer review history for this article is available at https://www.webofscience.com/api/gateway/wos/peer-review/10.1002/btm2.10625.

## Supporting information


**Table S1:** Specific search terms of databases.

## Data Availability

The data underlying this article are available in the article and in its online supplementary material.
