# Supplementary material for: Microfluidic chip as a promising evaluation method in assisted reproduction: A systematic review
Source: Bioeng Transl Med. 2023 Nov 24;9(2):e10625. doi: 10.1002/btm2.10625 (PMC10905557; doi:10.1002/btm2.10625)
Supplement: Supplementary file 1 — Table S1: Specific search terms of databases. [file BTM2-9-e10625-s001.docx]

| Supplementary Table 1. Specific search terms of databases. | | |
| --- | --- | --- |
| **Database** | **Search term** | **Number** |
| **Pubmed** | ((assisted reproductive technology) OR (in vitro fertilization) OR (clomiphene citrate) OR (superovulation) OR (gamete intrafallopian transfer) OR (in vivo fertilization) OR (zygote intrafallopian transfer) OR (artificial insemination) OR (cryopreservation) OR (intracytoplasmic sperm donation) OR (embryo) OR (oocyte) OR (sperm) OR (infertility therapy) OR (assisted reproduction)) AND ((microfluidic) OR (chip)) AND ((monitor) OR (assay) OR (sensor) OR (biochemical) OR (screen) OR (biomarker)) | 1821 |
| **Scopus** | TITLE-ABS-KEY ( "assisted reproductive technology" OR "in vitro fertilization" OR "clomiphene citrate" OR "superovulation " OR "gamete intrafallopian transfer" OR "in vivo fertilization" OR "zygote intrafallopian transfer" OR "artificial insemination" OR "cryopreservation" OR "intracytoplasmic sperm donation" OR "embryo" OR "oocyte" OR "sperm" OR "infertility therapy" OR "assisted reproduction") AND TITLE-ABS-KEY ( microfluidic OR chip ) AND TITLE-ABS-KEY (monitor OR assay OR sensor OR biochemical OR screen OR biomarker) | 1313 |
| **Web of Science** | TS=(assisted reproductive technology or in vitro fertilization or clomiphene citrate or superovulation or gamete intrafallopian transfer or in vivo fertilization or zygote intrafallopian transfer or artificial insemination or cryopreservation or intracytoplasmic sperm donation or embryo or oocyte or sperm or infertility therapy or assisted reproduction) AND TS=(microfluidic or chip) AND TS=(monitor or assay or sensor or biochemical or screen or biomarker) | 1985 |
| **ScienceDirect** | Title, abstract, keywords: (assisted reproductive technology OR in vitro fertilization OR clomiphene citrate OR superovulation OR gamete intrafallopian transfer OR in vivo fertilization OR zygote intrafallopian transfer OR artificial insemination OR cryopreservation OR intracytoplasmic sperm donation OR embryo OR oocyte OR sperm OR infertility therapy OR assisted reproduction) AND (microfluidic OR chip) AND (monitor OR assay OR sensor OR biochemical OR screen OR biomarker) | 151 |
| **IEEE Xplore** | ((( "assisted reproductive technology" OR "in vitro fertilization" OR "clomiphene citrate" OR "superovulation " OR "gamete intrafallopian transfer" OR "in vivo fertilization" OR "zygote intrafallopian transfer" OR "artificial insemination" OR "cryopreservation" OR "intracytoplasmic sperm donation" OR "embryo" OR "oocyte" OR "sperm" OR "infertility therapy" OR "assisted reproduction") AND ( microfluidic OR chip ) AND (monitor OR assay OR sensor OR biochemical OR screen OR biomarker))) | 70 |
